# Silencing Lysine-Specific Histone Demethylase 1 (LSD1) Causes Increased HP1-Positive Chromatin, Stimulation of DNA Repair Processes, and Dysregulation of Proliferation by Chk1 Phosphorylation in Human Endothelial Cells

**DOI:** 10.3390/cells8101212

**Published:** 2019-10-07

**Authors:** Martyna Wojtala, Arkadiusz Dąbek, Dorota Rybaczek, Agnieszka Śliwińska, Ewa Świderska, Katarzyna Słapek, Assam El-Osta, Aneta Balcerczyk

**Affiliations:** 1 Department of Molecular Biophysics, Faculty of Biology and Environmental Protection, University of Lodz, Pomorska 141/143, 90-236 Lodz, Poland; martyna.wojtala@biol.uni.lodz.pl (M.W.); arkadiusz.dabek@unilodz.eu (A.D.); 2 Department of Cytophysiology, Faculty of Biology and Environmental Protection, University of Lodz, Pomorska 141/143, 90-236 Lodz, Poland; dorota.rybaczek@biol.uni.lodz.pl; 3 Department of Nucleic Acids Biochemistry, Medical University of Lodz, Pomorska 251, 92-213 Lodz, Poland; agnieszka.sliwinska@umed.lodz.pl; 4 Department of Medical Biochemistry, Medical University of Lodz, Mazowiecka 6/8, 92-215 Lodz, Poland; ewa.swiderska@stud.umed.lodz.pl; 5 Scientific Student Circle of Young Biophysicists, University of Lodz, Pomorska 141/143, 90-236 Lodz, Poland; kasiaslapek@gmail.com; 6 Epigenetics in Human Health and Disease Laboratory, Department of Diabetes, Monash University, 99 Commercial Road, Alfred Centre, Melbourne 3004, Australia; sam.el-osta@monash.edu; 7 Hong Kong Institute of Diabetes and Obesity, Prince of Wales Hospital, The Chinese University of Hong Kong, Hong Kong

**Keywords:** LSD1, histone posttranslational modifications, cell cycle, Chk1, chromatin remodeling, DNA damage and repair

## Abstract

The methylation of histone lysine residues modifies chromatin conformation and regulates the expression of genes implicated in cell metabolism. Lysine-specific demethylase 1 (LSD1) is a flavin-dependent monoamine oxidase that can demethylate mono- and dimethylated histone lysines 4 and 9 (H3K4 and H3K9). The removal of methyl groups from the lysine residues of histone and non-histone proteins was found to be an important regulatory factor of cell proliferation. However, its role has not been fully elucidated. In this study, we assessed LSD1-mediated cell cycle progression using a human endothelial cell model. The short hairpin RNA knockdown of LSD1 inhibits the G_2_/M phase of cell cycle progression by checkpoint kinase 1 (Chk1) phosphorylation (S137). We observed elevated DNA damage, which was consistent with the increased detection of double-strand breaks as well as purines and pyrimidines oxidation, which accompanied the activation of ATR/ATRIP signaling by H2AXS139 phosphorylation. The irreversible pharmacological inhibition of LSD1 by 2-phenylcyclopropylamine (2-PCPA) inactivated its enzymatic activity, causing significant changes in heterochromatin and euchromatin conformation assessed by chromatin assembly factor 1 subunit A (CAF1A) and heterochromatin protein 1 isoform α and γ (HP1α/γ) immunofluorescence analysis. We conclude that the knockdown of LSD1 in endothelial cells leads to increased HP1-positive chromatin, the stimulation of DNA repair processes, and the dysregulation of proliferation machinery.

## 1. Introduction

Recent studies in transcriptional biology have highlighted the importance of epigenetic determinants in the regulation of cell metabolism. One of the main components of the regulatory machinery is the modulation of the demethylation of histone proteins by lysine-specific demethylase 1, LSD1 (also known as KDM1A, AOF2, and BHC110) [1]. LSD1 was the first of several protein lysine demethylases discovered [2], and to date, it is one of the most studied enzymes responsible for the demethylation of mono- and dimethylated lysines at position 4 or 9 in histone H3 (H3K4 and H3K9, respectively). The structure of LSD1 classifies the enzyme as a member of the monoamine oxidase (MAO) superfamily, and is highly conserved among many species, ranging from *Schizosaccharomyces pombe* to human [3,4,5].

The complexity of the demethylation process catalyzed by LSD1 depends on the interaction of the enzyme with specific chromatin regulatory complexes, including RE1-silencing transcription factor (REST), co-repressor CoREST (co-repressor for element 1-silencing transcription factor), nucleosome remodeling and histone deacetylation (NuRD), and SNAIL/Slug, or RCOR2 [6,7,8,9,10]. The broad consequences of demethylation controlled by LSD1 explain the substrate specificity of the lysine-specific demethylase containing histones as well as many crucial non-histone proteins, especially transcription factors (i.e., E2f1), chromatin-regulating proteins (i.e., Dnmt1), and also tumor suppressor proteins (i.e., p53) [11,12,13]. Additionally, the switching activity of LSD1 from a repressor function to that of a co-activator seems to be therapeutically attractive, and a major component in epigenetic reprogramming, as well as in the regulation of the cell cycle, which depends on a highly regulated series of converging signals including transcription factors, non-coding RNAs, DNA methylation, and histone modifications [14,15,16].

Recent findings provide more and more details into the epigenetic machinery picture that controls cell proliferation and survival [17,18,19]; nevertheless, the chromatin remodeling process that is important for cell cycle regulation and DNA damage response remain still incompletely understood. The interaction of co-activators and co-repressors with LSD1 plays a significant role in altering chromatin structure through the modification of core histone amino acid tails [20,21]. It was found that LSD1 is recruited to the chromatin of cells at G_1_/S/G_2_ phases [22], and its genetic ablation in embryonic stem cells (ESCs) results in the impaired differentiation properties of cells, apoptosis induction, and failure in maintaining global DNA methylation [12,23]. Conversely, a high overexpression of LSD1 in many solid tumors possessing aggressive clinicopathological features, i.e., neuroblastoma, chondrosarcoma, or hepatocellular carcinoma, suggests that the enzyme can serve as an oncogene in the context of malignant transformation [24,25,26].

Most of the studies characterizing the role of LSD1 in metabolism is related with cancer biology, and little is known about the role of LSD1 in endothelial cell proliferation, which is crucial for circulatory functions as well as for cancer progression and metastasis. In this article, we examine the role of lysine-specific demethylase 1 (LSD1) in the control of endothelial cell cycle, using (i) pharmacological and (ii) transcriptional models of inactivation of the enzyme activity (2-PCPA-treated human microvascular endothelial cells-1/human umbilical vein endothelial cells (HMEC-1/HUVECs) and pTRIPZ shRNA vector transfected HMEC-1, respectively). The studies performed on immortalized (HMEC-1) as well as primary cells (HUVECs) showed that LSD1 efficiently affects endothelial cells proliferation, presumably via Chk1, and involves the ATR/ATRIP signaling pathway, as a result of promoting the transient formation of repressive chromatin.

## 2. Materials and Methods

### 2.1. Cell Culture and Chemical Inhibitor Description

HMEC-1 (human microvascular endothelial cells-1) were obtained from the Center for Disease Control and Prevention, Emory University (Atlanta, GA, USA). Cells were cultured in MCDB 131 medium (Life Technologies, Carlsbad, California, USA) containing 10 ng/mL of epidermal growth factor (Millipore, Burlington, MA, USA), 10 mM glutamine (Invitrogen; Carlsbad, CA, USA), and 10% heat-inactivated fetal bovine serum (Life Technologies, Carlsbad, CA, USA). For HUVECs (human umbilical vein endothelial cells), the same cell culture conditions were applied. Cells were isolated form veins of freshly collected umbilical cords, by collagenase type II digestion, according to Jaffe’s protocol [27], and used for the experiments at passage 3-4. A permission for HUVEC’s isolation was obtained from the Bioethical Commission at Medical University of Lodz (decision no. RNN/264/15/KE).

2-PCPA, tranylcypromine hydrochloride, was purchased from Cayman Chemicals (Ann Arbor, MI, USA). 2-PCPA is an irreversible, mechanism-based inhibitor of LSD1 with an IC_50_ value of 20.7 µM and a K_i_ value of 242.7 µM that effectively inhibits histone demethylation in vivo. Although not as selective, 2-PCPA also irreversibly inhibits monoamine oxidase (MAO) type A and type B with IC_50_ values of 2.3 and 0.95 µM and K_i_ values of 101.9 and 16 µM, respectively [28,29].

### 2.2. shRNA Silencing of Lysine-Specific Histone Demethylase 1 (LSD1) Activity

The silencing of lysine demethylase-1 was performed in HMEC-1 using an inducible system for LSD1 knockdown (LSD1 KD) by shRNA (mature antisense sequence: 5′-GGAAAGAATCAAGGAGG-3′; Clone ID: V3THS_361041; Dharmacon; Lafayette, CO, USA). As a control for LSD1 DMT silencing, cells transduced with an empty vector were used: nonTarget (nonT; #RHS4743; Dharmacon; Lafayette, CO, USA). shRNA vector amplification in 293FT cells and the subsequent transduction of HMEC-1 was performed according to the manufacturer’s protocol. Upon the addition of 1 µg/mL doxycycline (DOX) to HMEC-1, to induce LSD1 silencing, expression of the targeted gene was downregulated by >70%, as measured by quantitative PCR. Changes in the expression of demethylase were also estimated by Western blotting at the protein level.

### 2.3. Immunocytochemistry

Immunocytochemical detection of both total and phosphorylated form of Chk1 was performed using rabbit polyclonal antibodies purchased from Abcam (Cambridge, UK) and Cell Signaling Technology (Danvers, MA, USA), respectively. Rabbit polyclonal antibodies specific to HP-1α and HP-1γ proteins were purchased from Abcam (Cambridge, UK). Rabbit polyclonal antibodies specific both to phospho-H2AX (i.e., H2AXS139ph) and ATRIP, rabbit monoclonal antibodies specific to CAF1A, as well as mouse monoclonal antibodies specific to PCNA were purchased from Cell Signaling Technology (Danvers, MA, USA). Bound primary antibodies were detected with secondary goat anti-rabbit Alexa Fluor 488-labeled antibody (Cell Signaling Technology, Danvers, MA, USA).

HMEC-1 were grown in 8-well tissue culture plates containing sterile coverslips, and were treated as indicated in the figure legends. For the immunocytochemical detection of total Chk1, phospho-Chk1 (Chk1S317ph), phospho-H2AX (H2AXS139ph), PCNA, ATRIP, HP-1α, and HP-1γ, cells were fixed for 45 min in 4% paraformaldehyde (PFA) buffered with phosphate-buffered saline (PBS), as previously described [18]. The cells were pre-treated in a blocking buffer (10% horse serum, 1% bovine serum albumin (BSA), 0.02% NaN_3_, 1x PBS) for 1 h at room temperature to minimize the non-specific adsorption of antibodies to the coverslips, and subsequently were incubated overnight at 4 °C with primary antibodies (1:750). Cells were washed three times (5 min each) with PBS/0.2% Triton X-100 (PBT) prior to incubation with secondary antibodies (1:1000) for 1 h at 37 °C in the dark. Next, cells were washed three times with PBT (5 min each), and then for 5 min in PBS. Coverslips were covered by cover glass under 4 μL of Vectashield mounting medium (Vector Laboratories) containing 4′,6-diamidyno-2-fenyloindol (DAPI; for Chk1 total, Chk1S317ph, PCNA, ATRIP, CHAF1A, HP1α and HP-1γ staining) or propidium iodide (PI; for H2AXS139ph staining). Observations were made using an AxioImager A1 fluorescence microscope (Zeiss, Jena, Germany) equipped with cyanine 3 (Cy3), green fluorescent protein (GFP), and DAPI filters. Negative control sections incubated with non-immune serum in the place of primary antibodies were free from immunostaining (data not shown); these negative control sections gave bright propidium iodide or DAPI signals, but completely lacked fluorescence in the wavelength of Alexa Fluor 488-conjugated secondary antibodies. Image data were collected at exactly the same exposure time on an AxioCam MRc5 CCD camera (Zeiss, Jena, Germany).

*Data collection for cell cycle analysis:* The quantification of aberrant mitoses (M-phase cells) was determined by counterstaining with DAPI (0.1 mg/mL; 4’,6-diamidino-2-phenylindole; Sigma-Aldrich, Saint Quentin, France) for 5 min at room temperature. The aberrant M-phase cells were observed using a fluorescence microscope equipped with UV-2A filter (UV-light; λ = 518 nm). All images were recorded at exactly the same time of integration with an AxioCam MRc5 CCD camera (Zeiss, Jena, Germany). The mitotic index was calculated as the percent ratio between the number of dividing cells and the entire HMEC-1 cell population. An index of aberrations (M-phase aberrant cells) was calculated as the percent ratio between the number of cells showing chromosome aberrations and all mitotic HMEC-1 cells. The apoptotic index was calculated as the percent ratio between the number of apoptotic cells and the entire HMEC-1 cell population.

### 2.4. Cell Cycle Analysis by FACS

*Cell preparation:* Cells were seeded onto the 6-cm dishes (NUNC™, Thermo Scientific™, Denmark) at a density of 40,000. After the incubation of cells with 2-PCPA or DOX (shRNA transfected cells) the medium was removed; then, cells were washed twice in PBS, trypsinized, washed twice in PBS, and centrifuged (3000 rpm, 3 min). After that, cells were fixed in 70% ethanol for 24 h at 4 °C. In the next step, the cell solution was centrifuged (10,000 rpm, 7 min), and the pellet was resuspended in PBS. Before analysis, cells were incubated for 30 min at 37 °C in the buffer containing RNase A (0.1 mg/mL; Sigma-Aldrich, St. Louis, MO, USA) and propidium iodide (PI, 40 µg/mL; Gibco™ Invitrogen™, Merelbeke, Belgium). Nuclear DNA content was measured using a LSRII flow cytometer and FACS Diva Software 6.2 (BD Biosciences).

*Measurement parameters:* The analysis of flow acquisitions was done using FlowJo software 10.4.1. For the identification of subpopulations of cells in different cell cycle phases, we used forward (FSC) and side scatter channels (SSC). In the first step of analysis, we removed the debris by performing an SSC-A versus FSC-A plot. After that, we used a FSC-H versus FSC-A plot to exclude clumps and doublets. To get rid of debris remains and some apoptotic cells, we used an SSC-H versus SSC-A plot. In the last step, gated cells were applied to the propidium iodide (PE-A versus PE-W). A final histogram plot was generated by using Count versus PE-A. The gating of cells was validated in the control sample, and the same parameters of gating were applied to the other samples. Further analysis of the identification of the subpopulation of cells in different phases of cell cycles was performed by a mathematical algorithm, which attempts to fit Gaussian curves to each phase (FlowJo Single Cell Analysis Software 10).

### 2.5. Total RNA Isolation, cDNA Synthesis, and Quantitative PCR

Total RNA was isolated using the total RNA Isolation System (InviTrap Spin Cell RNA Mini Kit, Stratec Molecular, Birkenfeld, Germany) according to the manufacturer’s recommendations. Genomic DNA was removed by using selective binding to a specific surface of solid material delivered by the kit producer (Stratec Molecular, Birkenfeld, Germany).

Reverse transcription reactions were performed on 2 μg of total RNA with the PrimeScript RT Master Mix (Perfect Real Time, Takara, Kusatsu, Shiga, Japan), following the manufacturer’s protocol.

Quantitative Real-Time PCR was performed using an Eco Real-Time PCR System (Illumina, San Diego, CA, USA). Total reaction volume (10 μL) was mixed using 0.2 nanomoles of forward and reverse primer, 1 µl of cDNA template (approximately 10 ng), and 5 µL of Takara BioSYBR Green Master Mix, according to the manufacturer’s recommendations. The amplification conditions for the Takara BioSYBR Green Master Mix Eco Real Time PCR (Illumina) consisted of an initial step of 30 s at 95 °C, followed by 40 cycles of 5 s at 95 °C, and 15 s at 60 °C. The following gene-specific primers have been used: *LSD1* f-TGGTAAGAGGTCTGGAGGGA, r-CAGCTTGTCCGTTGGCTTC; *CCNA2* f-ACTGGTGGTCTGTGTTCTGTGA, r-GATGCCAGTCTTACTCATAGCTGAC; *CCNB1* f-TGGTGAATGGACACCAAVTC, r-TAGCATGCTTCGATGTGGCA; *CCNE1* f-CAGGGAGCGGGATGCG, r-GGTCACGTTTGCCTTCCTCT; *PCNA* f-GCTCTTCCCTTACGCAAGTCT, r- AGTCTAGCTGGTTTCGGCTT; *p21* f-CCAGACCAGCATGACAGATTTC, r-GCTTCCTCTTGGAGCAGATCAG; and *P53* f-TCTGGGACTTAGTGCCTTTTATGG, r-CAGTCAGAAACTGTCAAATCATCCA. Gene expression levels were normalized to the level of *HPRT1* f-ATGGACAGGACTGAACGTCTT, r-TCCAGCAGGTCAGCAAAGAA. The delta–delta C_t_ method was used to determine the relative levels of mRNA expression between experimental samples and controls.

### 2.6. Western Blotting

Whole protein cell extract was prepared by using M-PER solution (Thermo Fisher Scientific, Waltham, MA, USA). First, 20 µg of proteins was separated by SDS-PAGE and transferred to a polyvinylidene difluoride (PVDF) membrane using a transfer apparatus according to the manufacturer’s protocols (Bio-Rad, Warsaw, Poland). After incubation with 5% non-fat milk in Tris-buffered saline with Tween 20 (TBST; 10 mM Tris, pH 8.0, 150 mM NaCl, 0.5% Tween 20) for 1 h, the membrane was washed three times for 5 min in TBST at room temperature. Next, the PVDF membrane was probed with primary antibodies: CAF1A (1:1000, Cell Signaling Technology, Baverly, MA, USA, Cat. 5480); ph-Chk1*(S317)* (1:1000, Cell Signaling Technology, Baverly, MA, USA, Cat. 12302); *Chk1* (1:1000, Abcam, ab47444); ph-H2AX(S139) (1:1000, Cell Signaling Technology, Baverly, MA, USA, Cat. 9718); HP1α (1:1000, Cell Signaling Technology, Beverly, MA, USA, Cat. 2616); and HP1γ (1:1000, Cell Signaling Technology, Beverly, MA, USA, Cat. 2619); PCNA (1:2000, Cell Signaling Technology, Beverly, MA, USA, Cat. 2586) was used as a loading control. The incubation was proceeded overnight at 4 °C with agitation. All primary antibodies were diluted in a 3% BSA solution. The next day, membranes were incubated with horseradish-peroxidase-conjugated secondary antibodies (1:2000, R&D Systems, Minneapolis, MN, USA, Cat. HAF007, Cat. HAF008) for 1.5 h at room temperature in agitation. The signal from membrane was visualized using enhanced chemiluminescence (ECL) solution (WESTAR ETA C 2.0, Cyanagen; Bologna, Italy) and charge-coupled devices (CCD) digital imaging system Alliance Mini HD4 (UVItec Limited, Cambridge, UK). Values for all analyzed protein were normalized to the loading control, PCNA.

### 2.7. Assessing of DNA Damage

*Alkaline comet assay*. Double DNA strand breaks in nonT/LSD1 KDs HMEC-1 were evaluated by comet assay and pulsed-field gel electrophoresis. The comet assay was performed under alkaline conditions essentially according to the procedure of Ahnström et al. [30]. A freshly prepared suspension of cells in 0.75% low-melting point (LMP) agarose in PBS was spread onto microscope slides pre-coated with 0.5% NMP agarose. Then, the cells were incubated for 1 h at 4 °C in a lysis buffer (2.5 M NaCl, 100 mM EDTA, 1% Triton X-100, 10 mM Tris, pH 10). After lysis, the slides were placed in an electrophoresis unit, and the DNA was allowed to unwind for 20 min in the electrophoresis solution (300 mM NaOH, 1 mM EDTA, pH >13). Electrophoresis was performed at 4 °C for 20 min at 0.73 V/cm (290 mA). Then, the slides were washed in water, drained, stained with DAPI (2 μg/mL), and covered with coverslips. To eliminate additional DNA damage, all the steps of protocol were performed under dimmed light or in the dark. The slides were observed at 200× magnification using an Eclipse fluorescence microscope (Nikon, Tokyo, Japan) attached to a COHU 4910 video camera (Cohu, Inc., San Diego, CA, USA) equipped with a UV-1 filter block consisting of an excitation filter (359 nm) and a barrier filter (461 nm) and image analysis system, Lucia-Comet v. 4.51 (Laboratory Imaging, Prague, Czech Republic). One hundred images were randomly selected from each sample, and the comet tail DNA (tail DNA [%]) was measured. Each experiment was repeated three times. The percentage tail DNA is positively correlated with the level of DNA breakage or/and the number of alkali-labile sites, and is negatively correlated with the level of DNA cross-links.

#### Repair Enzymed Modified Comet Assay

The alkaline comet assay using lesion-specific enzymes: endonuclease-III (Endo III) and formamidopyrimidine *N*-glycosylase (Fpg) were used to detect oxidized purines and pyrimidines (that are generated as a result of oxidative stress-induced DNA damage) [31,32]. The cell-agarose suspension slides were prepared as described above for the standard comet assay. After lysing, the slides were washed three times with the enzyme buffer (40 mM HEPES, 100 mM KCl, 0.5 mM EDTA, and 0.2 mg/mL bovine serum albumin) at room temperature, and were incubated at 37 °C for 30 min with (i) Endo III (1:1000, 30 min), (ii) Fpg (1:1000, 45 min), and (iii) with enzyme buffer (control). Endo III recognizes oxidized pyrimidines, while Fpg recognizes oxidized purines, specifically 8-oxo-guanine. The slides were coded and placed in a specifically designed horizontal electrophoresis tank, and the DNA was allowed to unwind for 20 min in alkaline solution containing 300 mM NaOH and 1 mM EDTA, pH >13. The DNA was electrophoresed for 30 min at 300 mA and 20 V (0.70 V/cm). Then, the slides were neutralized with 0.4 M Tris (pH 7.5), stained with SYBR Green I (1:10,000) for 1 h, and covered with coverslips. As above, 100 images were randomly selected from each sample, and the comet tail DNA (tail DNA (%)) was measured.

### 2.8. Statistical Analysis

Statistical analyses were performed by means of STATISTICA 8.0 PL software (StatSoft INC, Tulsa, Oklahoma). All data were expressed as mean ± SD. Differences between groups were assessed by the one-way ANOVA, and post hoc analysis by Tukey’s test was performed. A probability *p* < 0.05 was considered as statistically significant.

## 3. Results

### 3.1. Silencing of LSD1 Results In Abberations of Cell Cycle of Entothelial Cells

**Figure 1:**
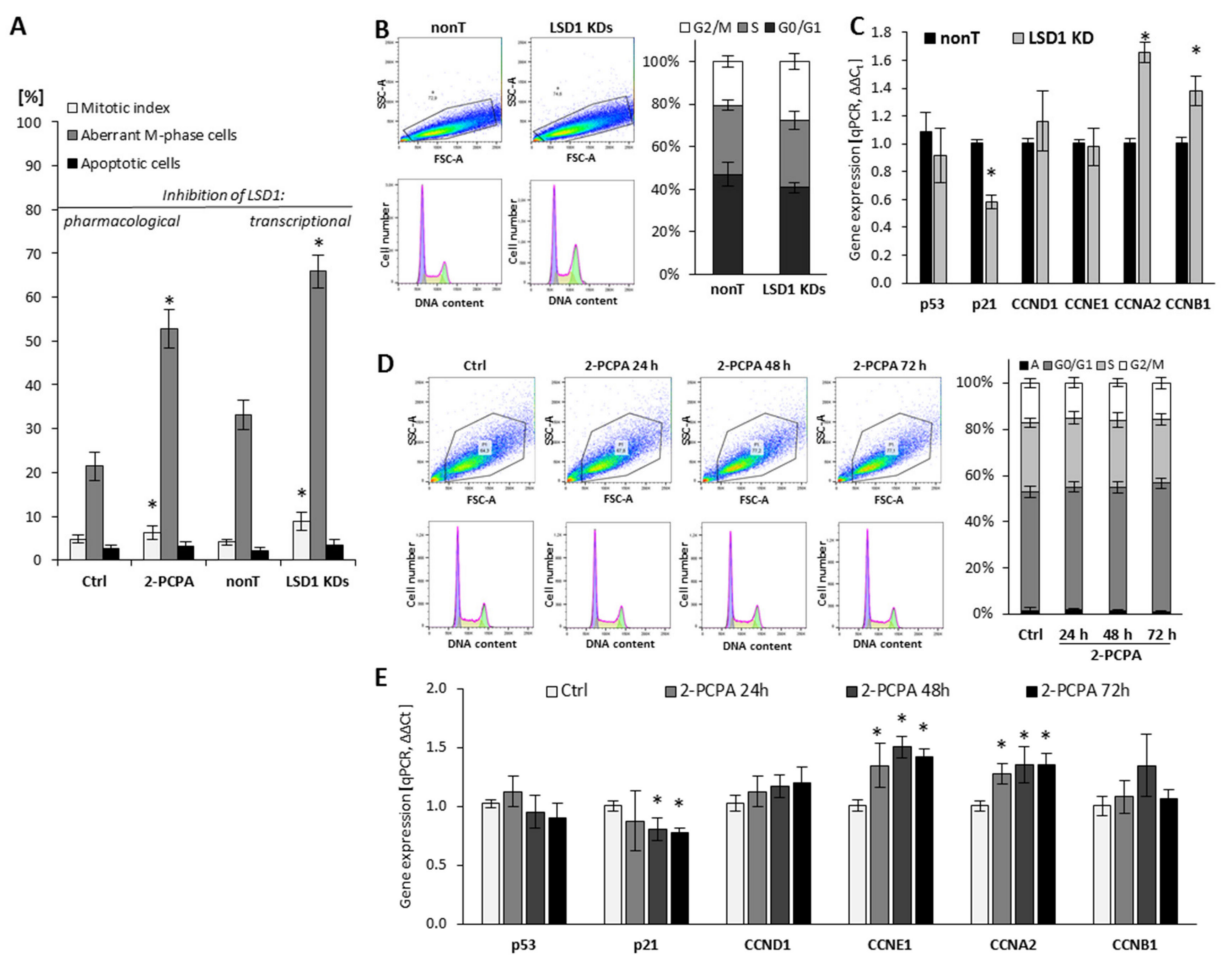
Effect of pharmacological and transcriptional inhibition of lysine-specific demethylase 1 (LSD1) on cell cycle progression of human microvascular endothelial cells (HMEC-1). (**A**) The effect of LSD1 shutdown due to 2-phenylcyclopropylamine (2-PCPA) treatment or shRNA transfection on the mitotic index, the induction of aberrations in the M phase of cell cycle and apoptosis (MI, a percentage of mitotic cells in relation to all cells, both interphase and mitotic). The percentages were calculated based on 3000 cells per repeat; (**B**,**D**) Flow cytometry analysis of cell cycle. Cells were stained with propidium iodide. The obtained histograms were analyzed using FlowJo software 10.4.1. (“A” stands for the subpopulation of apoptotic cells). Presented histograms are representative of three independent experiments; (**C**,**E**) Gene expression profile of cell cycle regulators in HMEC-1 treated with 100 µM of 2-PCPA (**E**) or after transcriptional LSD1 silencing by specific shRNA (**C**). Presented data are average of three independent experiments. The level of significance was determined at * *p* < 0.05, ANOVA and post hoc analysis by Tukey’s test.

The role of LSD1 in the regulation of endothelial cell proliferation was assessed using (i) flow cytometry for visualization of the cell cycle, (ii) fluorescent microscopy analysis for mitotic index calculation, and (iii) RT-qPCR for assessing the expression of genes implicated in the regulation of proliferation, as shown in Figure 1. The knockdown of LSD1 by shRNA (Figure S1) resulted in a significant modification of cell cycle progression, by increasing the population of cells in G_2_/M phase from 20% to 26–30%, as shown in Figure 1B. Due to the silencing of LSD1, we also observed changes in the expression of major cell cycle drivers such as downregulation of p21 and upregulation of cyclin A (CCNA2) and cyclin B (CCNB1) Figure 1C (* *p* < 0.05, ANOVA test). The expression of p53 and CCND1 was at the control level. Analysis of the mitotic index parameter (MI), which presents the number of mitotic cells in relation to all cells, confirmed the aberration of cell cycle related with shutting down LSD1 activity. Specifically, the fluorescent staining of cells (DAPI) showed increased MI in the population of cells deprived of LSD1 expression (LSD1 KDs HMEC-1) in comparison to control cells (nonT HMEC-1). Additionally, further microscopic analysis of the M phase of LSD1 KDs/nonT HMEC-1 revealed an increased number of LSD1 knockdown cells presenting abnormalities in the division phase, as shown in Figure 1A (grey bars on the figure presents the population of cells with aberrant M phase in relation to all mitotic cells). The aberrant M-phase cells showed pulverization (included chromosomal breaks and gaps (≤20), lost and lagging chromosomes, acentric fragments, segregation defects, and chromosome bridges). The representative visualization of the normal and aberrant M-cells is presented in Figure S2.

To confirm the data obtained on the LSD1 KDs model, we also used 2-PCPA (tranylcypromine chloride), a pharmacological inhibitor of monoamine oxidases, including lysine-specific demethylase 1 [28,29]. Pharmacological exposure of microvascular endothelial cells with the 2-PCPA (100 µM) for 24 h, 48 h, or 72 h did not the change cell cycle progression assessed by flow cytometry (Figure 1D). However, we observed slight changes in the expression of genes important for the cell proliferation process, including p21, cyclin E, and cyclin A (Figure 1E). Similarly to the population of LSD1 KDs HMEC-1, we found an increase of the MI in the 2-PCPA-treated cells, as shown in Figure 1A (left panel).

### 3.2. Silencing of LSD1 Activates Checkpoint Kinase 1 (Chk1)

**Figure 2:**
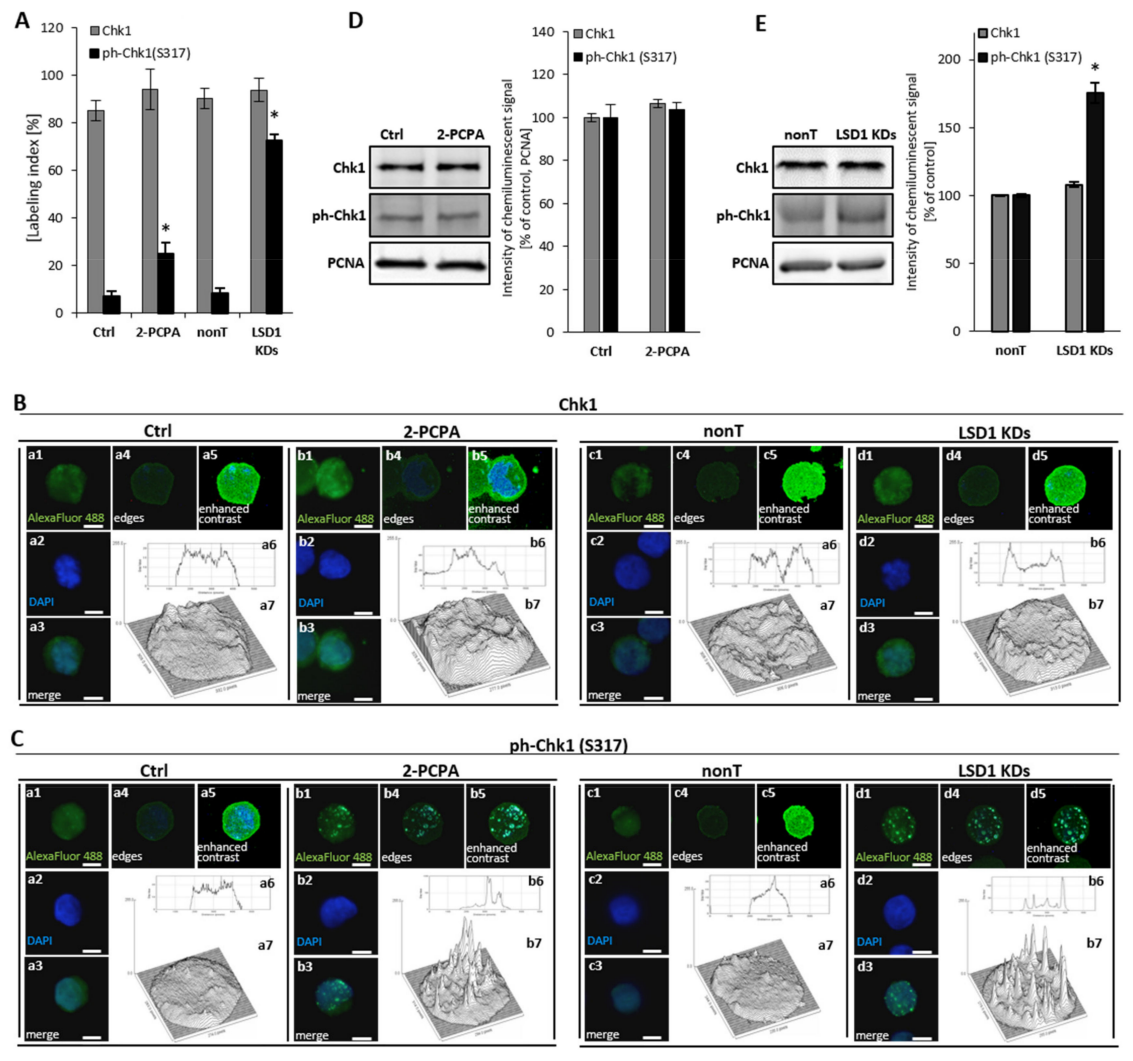
Consequences of LSD1 inhibition/silencing in human microvascular endothelial cells on checkpoint kinase 1 (Chk1) activation. Immunofluorescent analysis of the Chk1 level (**A**,**B**) and the Chk1 activation (ph-Chk1(S317) level (**A**,**C**). Cells were stained with anti-Chk1 or anti-ph-Chk1(S317) antibodies conjugated with AlexaFluor 488 fluorescent dye. The labeling index was calculated as the ratio of immunofluorescence-labeled cells to all cells in an HMEC-1 cell population. The intensity of fluorescence was visualized in ImageJ program (**B** a6, a7, b6, b7; **C** a6, a7, b6, b7). The presented pictures are representative of three independent experiments. (**D**), (**E**) Analysis at the protein level of the expression of Chk1 and ph-Chk1 in (**D**) pharmacological and (**E**) transcriptional experimental models of LSD1 activity inhibition. Scale bars are equal to 10 µm. Statistical significance was analyzed at * *p* < 0.05 using ANOVA and post hoc analysis by Tukey’s test.

Due to identified abnormalities in the cell cycle after shutting down of the LSD1 demethylase, including aberration of the M phase and changes in the mRNA expression of cell cycle drivers (cyclin B, cyclin A, p21), as shown in Figure 1, we next analyzed the activation state of checkpoint kinase 1 (Chk1), which is critical cycline-dependent kinase (CDK) for G_2_/M phase transition [33]. Immunofluorescent staining of the phosphorylated form of checkpoint kinase 1 (ph-Chk1(S317)) clearly showed an increased level of the activated protein in the knockdown cells, as shown in Figure 2A (black bars). These data were confirmed by protein blot of whole cellular lysates, as shown in Figure 2E. We did not find changes between nonT HMEC-1 and LSD1 KDs HMEC-1 in the level of unmodified Chk1, as shown in Figure 2A (grey bars) and Figure 2E. For better visualization of the immunofluorescence signal, we used ImageJ software, which allowed presenting the intensity of the fluorescence inside single nuclei, Figure 2B,C, and featured specific foci (images a6, a7, b6, and b7).

Furthermore, we observed the activation of Chk1 (ph-Chk1(S317)) in a pharmacological model, Figure 2A, that corresponds with earlier observations using LSD1 knockdown cells. However, the activation of Chk1 protein in 2-PCPA treated HMEC-1 was registered only by immunofluorescent analysis, as shown in Figure 2A,C, not by Western blotting, as shown in Figure 2D. Primary HUVECs responded to 100 µM of 2-PCPA by the strong activation of Chk1, Figure S3, comparable to LSD1 knockdown cells.

Based on the obtained results from analysis of cell cycle and Chk1 activation, we concluded that the silencing of LSD1 results in the G_2_/M cell cycle arrest.

### 3.3. LSD1 Alters Chromatin Conformation

As LSD1 activity decides the histone H3 transcription activation/repression marks [24,26], we performed an analysis of the expression and localization of structural proteins involved in chromatin organization such as HP1 proteins, chromatin assembly factor 1 (CAF1), and proliferating cell nuclear antigen (PCNA). All the indicated molecules define a specific architecture for replication foci at pericentric heterochromatin.

**Figure 3:**
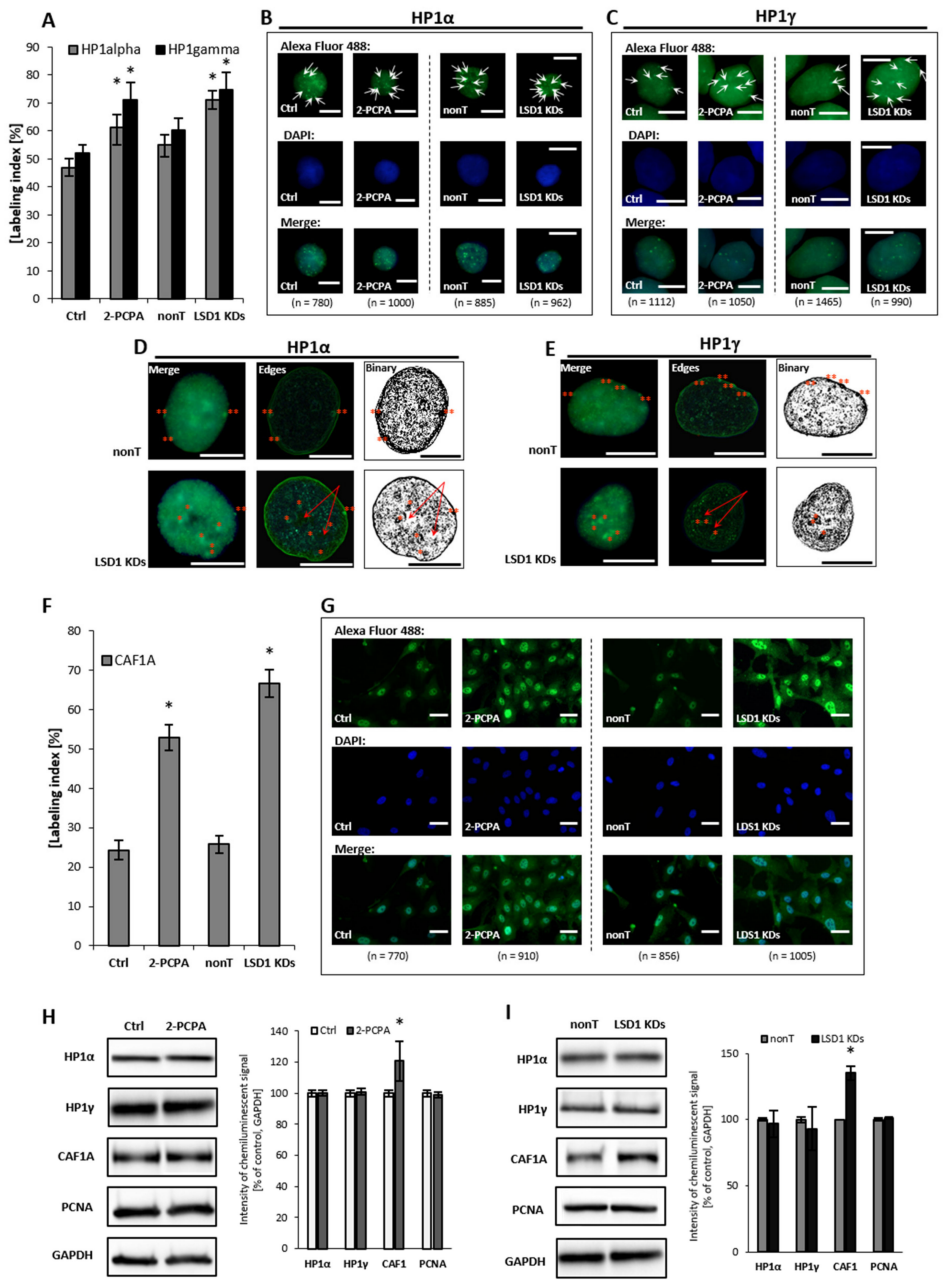
Effect of pharmacological and transcriptional inhibition of lysine-specific demethylase 1 (LSD1) on the ‘topography’ of heterochromatin fraction. Immunofluorescent analysis of HP1 alpha and gamma localization (**A**–**E**) and CAF1A protein (**F**,**G**). The labeling index was calculated as the ratio of immunofluorescence-labeled cells to all cells in an HMEC-1 cell population. Subfigures D and E show exemplary nuclei from the series “not-treated” (nonT) versus “treated” (LSD1 KDs) for respectively: HP1 alpha protein and HP1 gamma, and enroll the most characteristic location of specific foci, labeling heterochromatin regions at the periphery of the interphase nuclei (double red star), which is usually associated with the nuclear lamina, and perinucleolar heterochromatin (single red star). The red arrows indicate the unlabeled region (the so-called “hole”) in the location of the nucleoli. The protein expression of HP1 aplha, HP1 gamma, and CAF1A by Western blotting in cells treated with 100 µM of 2-PCPA (**H**), and (**I**) shRNA-transfected HMEC-1. Presented images are representative, and the data are the average of three independent experiments. Scale bars are equal to 10 µm. * *p* < 0.05, ANOVA and post hoc analysis by Tukey’s test.

HP1 protein, and its α and γ isoforms, mediate heterochromatin formation and are implicated in gene silencing by binding to methylated lysines of histone 3 and 4 (H3, H4) i.e., K9, K27, K36, K79, and K20, which are recognized as the repressive marks, and promoting the recruitment of other silencing factors, including DNA methyltransferases. The proteins, predominantly localized in the heterochromatin—except for HP1γ, which is found both in euchromatin and heterochromatin fractions—also participate in telomeric chromatin organization, and are crucial determinants of genome stability [34,35]. In our experimental model, we found that the silencing of LSD1 by shRNA and pharmacological inhibition, reorganizes HP1 to heterochromatic sites and increases HP1α and HP1γ content by about 20% to 35% when compared to the adequate controls, Figure 3A–C. Detailed microscopic analysis of images after immunofluorescent staining show significantly larger HP1α-enriched clusters at the heterochromatin–euchromatin boundaries in LSD1 KD cells and in cells after 2-PCPA treatment, as shown in Figure 3D and Figure S4. These changes might lead to an impaired replication of heterochromatic sequences, and we suggest the engagement of the Chk1-dependent pathway in the LSD1-dependent cell cycle progression. Changes that were registered by immunofluorescent analysis were not confirmed by Western blotting, as shown in Figure 3H,I. The differences between the labeling indices of control and inhibitor/shRNA samples were about 20%, as shown in Figure 3A, and we assume that it might be below the detection sensitivity of the Western blotting (WB) technique.

Human CAF1 is a nucleosome assembly factor that deposits newly synthesized and acetylated histones H3 and H4 into nascent chromatin during DNA replication. Similar to HP1γ, CAF1 is targeted to heterochromatic and euchromatic DNA replication foci fractions. Looking for the effect of LSD1 on dynamics in heterochromatin fraction, we found a significant increase in the amount of CAF1A (a 150-kDa subunit of the CAF1 complex) in LSD1 KDs, as well as in LSD1 inhibitor-treated cells, as shown in Figure 3F–I and Figure S5. CAF1A (CAF1-p150) is targeting the CAF1 complex to the replication fork through direct interaction with proliferating cell nuclear antigen (PCNA). As we found an increased level of CAF1-p150, we decided to check the PCNA protein level; nevertheless, we did not observed changes in the cellular content of protein neither in HMEC-1 (Figure 3H,I), nor in the HUVEC experimental model Figure S5.

In conclusion, the performed studies revealed the potential of LSD1 to affect the higher-order chromatin structure. The identified changes in the expression and localization of the chromatin dynamics marks, especially HP1α, suggest that LSD-1 acts as a chromatin relaxing agent.

### 3.4. Induction of DNA Damage Corresponds with Decreased LSD1 Activity

Due to the significant modifications of the heterochromatin fraction, we assessed potential DNA damage level using an alkaline comet assay, with and without the addition of the repair enzymes: endonuclease-III (Endo III) and formamidopyrimidine *N*-glycosylase (Fpg). Formamidopyrimidine DNA glycosylase (Fpg) plays a crucial role in the first step of base excision repair, and removes mainly 7,8-dihydro-8-oxo-2’deoxyguanine (8oxoG) and 2,6-diamino-4-hydroxy-5-*N*-methyl formamidopyrimidine from DNA, leading to the generation of apurinic/apyrimidinic sites [31,32]. Endo III (Nth) is a restriction endonuclease that is responsible for the identification of oxidized pyrimidines and their transformation into DNA strand breaks.

**Figure 4:**
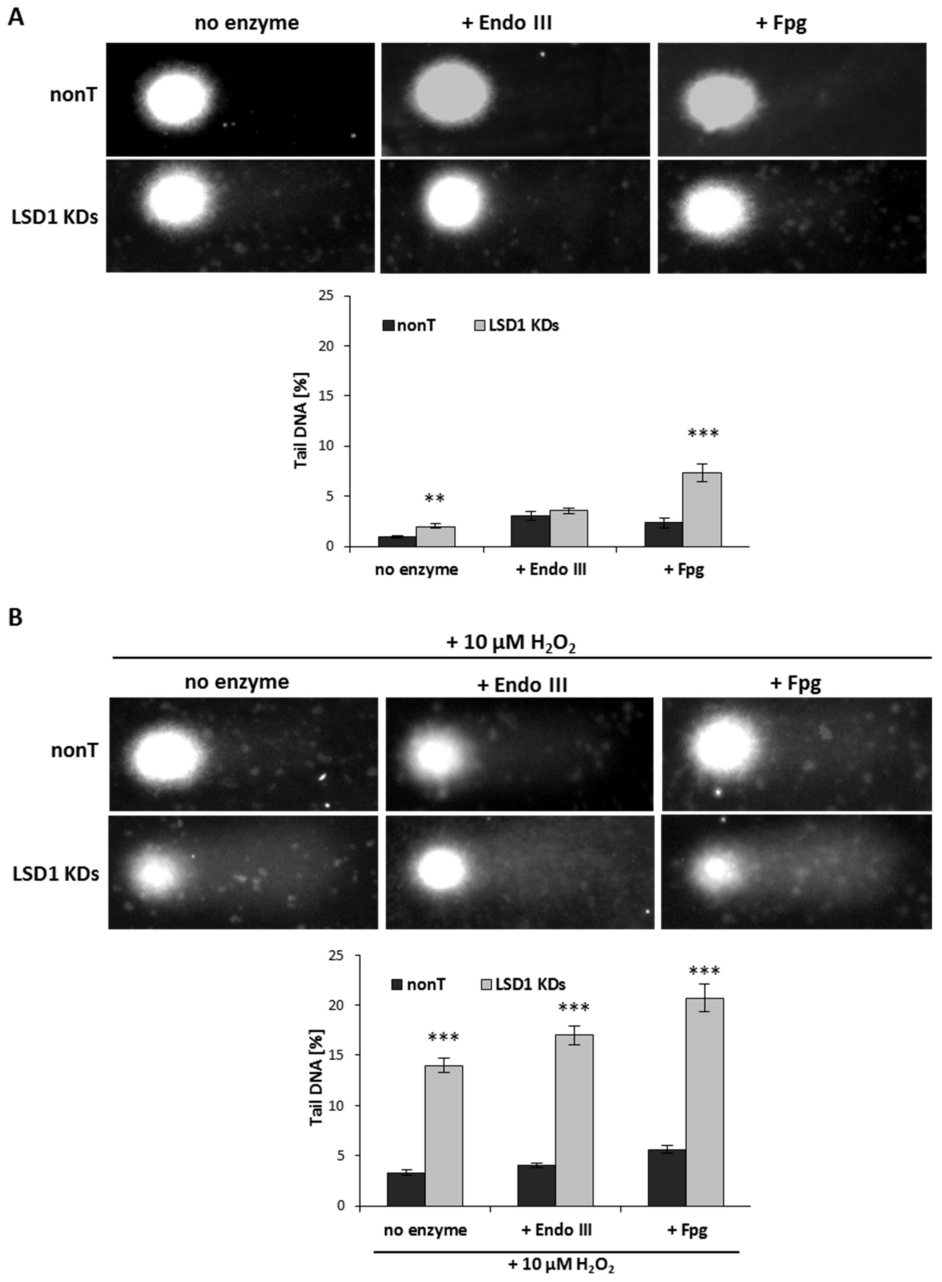
The tail moment (TM) of oxidatively damaged DNA in the nonT and LSD1 KDs HMEC-1 evaluated by alkaline and modified comet assay using lesion-specific enzyme endo III (endonuclease III) and FPG (formamidopyrimidine glycosylase) to differentiate DNA double-strand breaks (cells untreated) from oxidized purines (+ endo III) and pirymidines (+ Fpg). (**A**), (**B**) All the values are expressed as mean ± SD (the number of cells used per repeat, *n* = 100). The level of significance was determined at *** *p* < 0.001 and ** *p* < 0.01 nonT vs. LSD1 KDs (ANOVA and post hoc analysis by Tukey’s test).

We observed that LSD1 shRNA cells are more susceptible to DNA damage when compared to nonT control HMEC-1, Figure 4A (no enzyme samples, alkaline unmodified comet assay).

Next, we induced oxidative stress using 10 µM of hydrogen peroxide, and observed elevated DNA damage (seven times higher when compared to untreated LSD1 KD cells), as shown in Figure 4B versus Figure 4A (no enzyme samples). In close agreement with the observation that enzyme-sensitive sites might be more prone for the strand breaks, we registered significantly higher DNA damage level (more than three times) in LSD1 KDs in a modified comet assay (with an Fpg repair enzyme that identifies oxidized pyrimidines), as compared to alkaline comet assay, as shown in Figure 4A. No changes in the percent tail DNA in the assay with EndoIII were observed between nonT and LSD1 KDs, as shown in Figure 4B. The level of purine and pyrimidine oxidation after H_2_O_2_ treatment was significantly increased when compared to nonT, as shown in Figure 4B.

**Figure 5:**
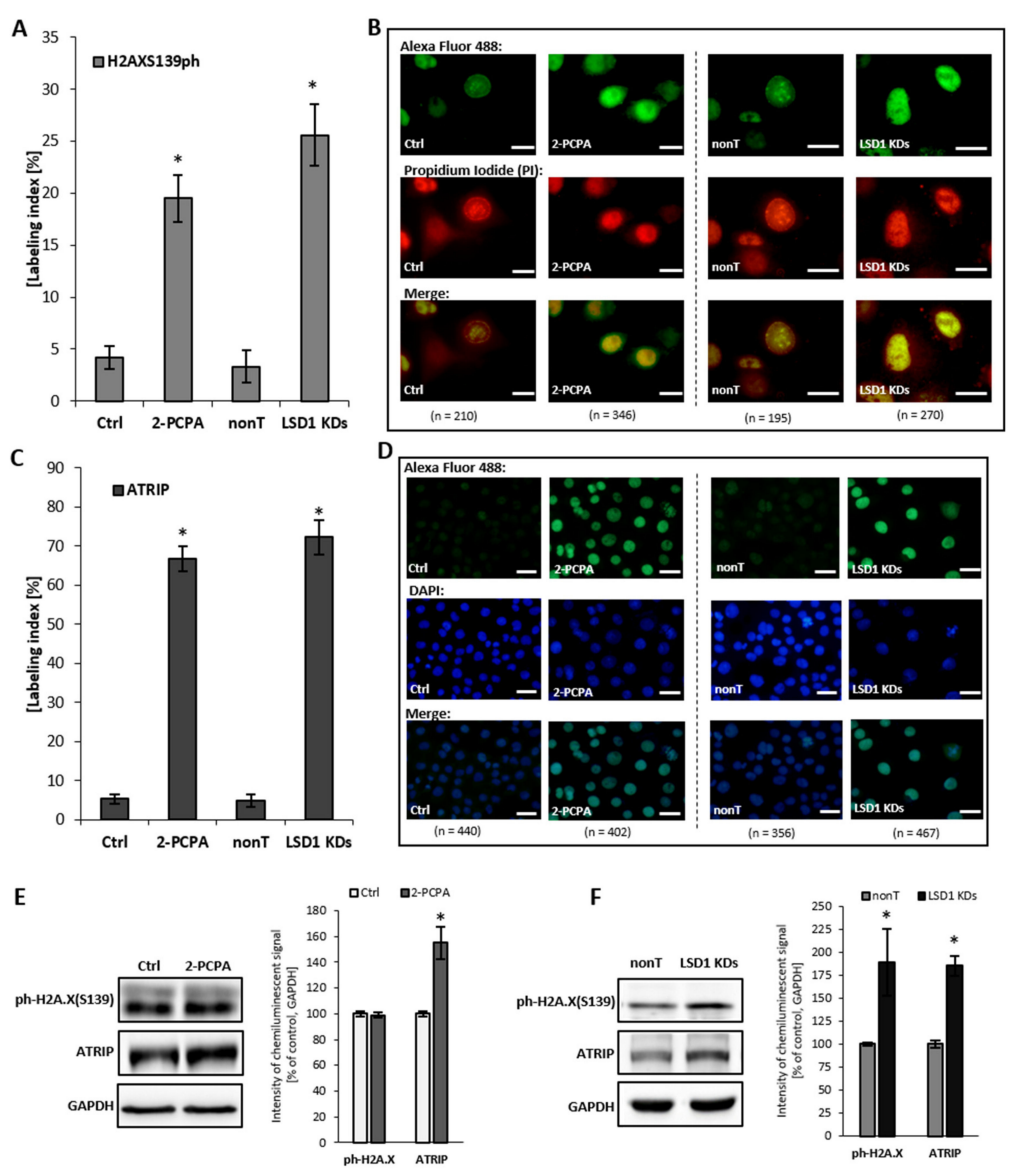
Level of DNA damage markers: H2AXS139ph and ATRIP after shutting down LSD1 activity. (**A**–**D**) immunofluorescent localization of the antigens, (**E**,**F**) protein level of markers by Western blotting. Presented images are representative, and the data are the average of three independent experiments. The labeling index was calculated as the ratio of immunofluorescence-labeled cells to all cells in an HMEC-1 cell population. Scale bars are equal to 10 µm. * *p* < 0.05, ANOVA and post hoc analysis by Tukey’s test.

To confirm the comet assay result, additionally, we assessed the level of expression of H2AX Ser139ph and ATRIP, which are the molecules indicated in the literature as markers for DNA damage, but also indirectly for DNA replication stress. We found that an elevated level of ATRIP in LSD1 KD cells closely corresponds with the pharmacological inhibition of the enzyme, as shown in Figure 5C–F. ATRIP and its partner, the master checkpoint kinase ATR, exist as a complex and function together as the DNA damage response. We observed also an increase in the phosphorylated histone H2AX Ser139 (Figure 5A,B,F), which is a sensitive and specific marker of the DNA damage, especially double-strand breaks (DBSs) and the subsequent repair of the DNA lesion. Altogether, the obtained data clearly show how critical the role of LSD1 is in the DNA repair processes.

## 4. Discussion

Lysine methylation patterns catalyzed by histone methyltransferases/demethylases (HMTs/HDMs) activity can regulate the cellular processes of diverse substrates such as histone and non-histone proteins, DNA, RNA, and lipids [36,37]. Through the intense development of pharmacoepigenetics, we are able to reverse many epigenetic modifications and support the treatment of various diseases, including cancer [38,39]. A number of studies focus on lysine-specific demethylase 1 (LSD1), because of its important role in the regulation of cellular metabolism. Increased LSD1 expression has been found in oral and breast cancers, as well as endometrioid endometrial adenocarcinoma [40,41,42].

In the presented study, we examined endothelial cell proliferation, mainly microvascular but also vein-origin endothelial cells (ECs) via the perspective of LSD1. ECs play a pivotal role in tumor growth and metastasis due to diapedesis process and their bridge function based on oxygen and nutrients delivery, which are necessary for tumor development. LSD1 serves as a double-edged sword by removing methyl groups from K4me1/me2 and/or K9me1/me2 of H3, thus affecting chromatin conformation and either promoting or repressing the transcription process [2,3]. Using experimental models of the pharmacological and genetic knockdown of the enzyme, we show a close correspondence for LSD1 in the regulation of chromatin topography, cell proliferation, and DNA damage/repair processes in endothelial cells. We found that shRNA silencing of LSD1 results in (i) an increase of the mitotic index (Figure 1A), and (ii) a block of the cell cycle in the G_2_/M phase confirmed by flow cytometry assay and gene expression level of cyclins, cyclin-dependent kinase (CDK) inhibitor p21 (Figure 1B,C), and Chk1 activation (Figure 2). At the same time, microscopic analysis revealed a significant increase of aberrant M-phase cells—both 2-PCPA treated and after shRNA LSD-1 silencing—and no change in the number of apoptotic cells (Figure 1A). Chk1 activation was confirmed by Western blotting, as well as by the immunofluorescence staining of ph-Chk1 (S317) (Figure 2). These observations are in contrast to our previous report on G9a histone methyltransferase (G9a HMT), which is an enzyme presenting a partially adverse effect to LSD1 due to the mono- and dimethylation of H3K9 (H3K9me1/me2), where we revealed that the silencing of the HMT blocks the cell cycle of endothelial cells in the G_0_/G_1_ phase [43]. However, based on the available literature, it appears that LSD1 cell cycle regulation can be cell-type specific. It was found that in acute lymphoblastic leukemia cell line MOLT-4 and mantle cell lymphoma JeKo-1, the downregulation of LSD1 by siRNA results in blocking the cell cycle in the G_0_/G_1_ phase, an increased expression of p21, and an induction of apoptosis [44]. In the human acute myeloid leukemia (AML) induced by the MLL-AF9 oncogene (MLL-AF9) mouse model, the abrogation of LSD1 resulted in a heightened rate of apoptosis and impaired leukemogenicity [45]. In addition, analysis performed on the cells from solid tumors, including prostate cancer cells (PC3 and DU145), showed the same cell cycle response scheme for LSD1 inhibition. Cells treated with HCI-2509, a reversible LSD1 inhibitor, responded with cell cycle block in the G_0_/G_1_ phase [46]. These may suggest that the inhibition of LSD1 in tumor cells results in G_0_/G_1_ cell cycle arrest and apoptosis induction, but recent data on clear cell renal cell carcinomas (ccRCC) and neuroblastoma cells show that such a generalization is exaggerated [47,48]. LSD1 inhibition by siRNA knockdown or using the small molecule inhibitor SP2509 suppressed the growth of ccRCC in vitro and in vivo in G_1_/S. In addition, a decreased H3K4 demethylation at the *CDKN1A* gene promoter was found, in association with p21 upregulation [47]. Whereas in neuroblastoma cells (NGP, LAN5 and SK-N-SH), the inhibition of LSD1 with HCI-2509 resulted in G_2_/M phase cell cycle arrest and an inhibitor concentration-dependent increase in the methylation of both H3K4me2 and H3K9me2 marks [48]. This is in line with the results presented here on the endothelial cell model; however, in the analyzed neuroblastoma cells, an increased level of p53 gene expression was observed, which we have not seen in our study.

The observed cell cycle disorder due to LSD1 impaired activity was accompanied by numerous changes in chromatin remodeling identified by HP1 and CAF1A immunostaining analysis. Mechanisms contributing to the maintenance of chromatin remodeling in proliferating cells remain poorly understood. One of the important elements characterizing proliferation machinery is the replication of heterochromatin occurring in the pericentric regions of chromosomes. In our experimental model, the immunofluorescence analysis showed that both the pharmacological and transcriptional silencing of LSD1 results in a significant increase of the heterochromatin protein 1 level, the α and γ isoforms (Figure 3A–C), and CAF1A protein (Figure 3F,G). Additionally, it was found that the shRNA downregulation of LSD1 expression is also associated with the perinucleolar heterochromatin localization of HP-1α and γ (Figure 3D,E, Figure S4). HP-1 proteins are key structural components of mitotic chromatin that ensure the integrity of chromosomes during cell division [49], and gene knockdown of HP-1 by RNAi in *Drosophila melanogaster* Kc cells have been shown to alter cell cycle progression, with a loss of S and G_2_/M cell populations and the accumulation of apoptotic cells [50]. The presented endothelial cell model confirms the interaction of HP1 with cell cycle progression also in a LSD1-dependent manner. An increased level of the other mark of heterochromatin dynamics—CAF1A, which was reported to function in a complex with HP1α and HP1γ proteins, but also newly synthesized histones H3 and H4 [51,52]—suggests that LSD1 is required for the stable maintenance of HP1α and HP1γ proteins at sites of pericentric heterochromatin, as it has occurred for Suv39h and H3K9 methyltransferase [53]. However, the molecular link between heterochromatin formation and the HP1<>CAF1<>methylation status of H3K4/H3K9 remains still unclear, but slightly better understandable due to the findings of Yang and co-workers [54]. They have identified in a fertility screen in female *Drosophila melanogaster* an *ovaries absent* gene (*ova*), which functions in the stem cell niche, downstream of P-element Induced WImpy testis (Piwi) protein, to support germline stem cell differentiation. Detailed analysis have revealed that *ova* links the H3K4 demethylase (dLSD1) to HP1α for local histone modifications during HP1α-mediated gene silencing, which is required for ovary development, transposon silencing, and heterochromatin formation [54].

The registered block of the cell cycle in the G_2_/M phase due to LSD1 silencing accompanied DNA damage and replication stress identified by comet assay and visualization of ATRIP and H2AX139ph levels (Figure 4, Figure 5). We also observed significant increases in oxidative DNA damage, twice as much in LSD1 KDs without oxidative stimulation of cells with hydrogen peroxide (Figure 4A) and four times more after the oxidant treatment of LSD1 KDs (versus adequate control, nonT; Figure 4B). These confirms the importance of the LSD1 in the DNA repair [55,56] also in ECs. Except for numerous irregularities identified by comet assay and Fpg glycosylase/EndoIII endonuclease, also other marks of DNA damage were significantly increased, i.e., H2AX139ph, confirming the existence of double-strand breaks (Figure 5A). The literature evidence suggesting that DNA double-strand breaks promote the transient formation of repressive chromatin [56] enhances our finding on the shRNA shutting down LSD1 activity. The presented results unambiguously show the importance of LSD1 in the regulation of the cell cycle, chromatin topography, and DNA repair process in endothelial cells, based on LSD1-impaired activity models, but also support the multifunctional LSD1 activity profile.

## 5. Conclusions

**Scheme 1:**
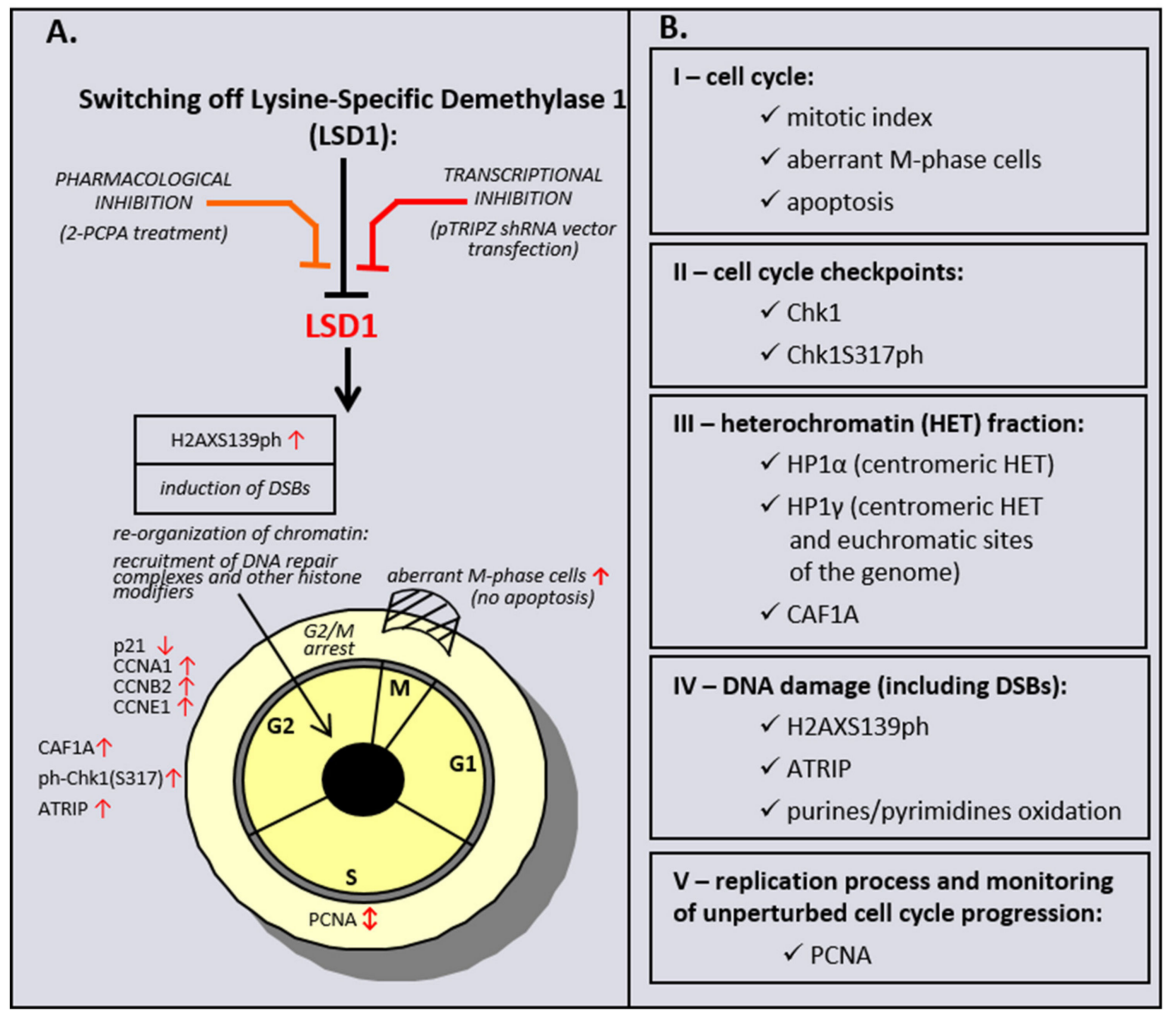
Effect of pharmacological and transcriptional inhibition of lysine-specific demethylase 1 (LSD1) on induction of the cell cycle and DNA damage response (DDR) pathway in microvascular endothelial cells (HMEC-1).

Taken together, the data obtained from a genetic inhibition of LSD1 HMEC-1, but also HMEC-1 and HUVECs treated with LSD1 inhibitor to a significant level, revealed a commonality of action between the chemically induced inhibition of LSD1 and its transcriptional repression, as schematically summarized in Scheme 1. The observed discrepancy between two models is probably related with specificity of the studied inhibitor. 2-PCPA beside LSD1 also irreversibly inhibits monoamine oxidases A and B (MAO-A, MAO-B), which are structurally related to the demethylase. A recent meta-analysis has shown that the overexpression of LSD1 is associated with worse prognosis in cancer patients [57]. Based on the obtained results, we hypothesize that LSD1 activity could be an important target for the anticancer therapy, as well as for the treatment of other diseases where endothelial dysfunction is observed, as our data confirm the dramatic importance of the demethylase in cell cycle progression and DNA repair, and show that the response of cells to LSD1 inhibition depends on the cell type origin.

## Supplementary Materials

The following are available online at https://www.mdpi.com/2073-4409/8/10/1212/s1, Figure S1: Expression level of LSD1 at the protein (upper images, charts; A, B) and transcript level (bottom graphs; C, D), modified by 2-PCPA treatment and due to shRNA silencing. title, Figure S2: Visualization of cells in different phases of mitosis (A) and cells undergoing apoptosis (B) after LSD1 shutting down. Figure S3: Effect of treatment of HUVECs with 2-PCPA on Chk1 activation. Figure S4: Visualisation of changes in the eu/heterochromatin fraction in HMEC-1 LSD1 KDs based on the HP1 alpha immunofluorescent staining. Figure S5: Effect of 2-PCPA on heterochromatin fraction remodeling in HUVECs. Figure S6: Effect of 2-PCPA on DNA damage indicators in HUVECs.

## List of Abbreviations

ATM	ataxia telangiectasia mutated serine/threonine kinase
ATR	ATM and Rad3-related checkpoint kinase
ATRIP	ATR-interacting protein
CAF1	chromatin assembly factor 1
Chk1	checkpoint kinase 1
DOX	doxocycline
HMEC-1	human microvascular endothelial cells
HMTs	histone methyltransferases
HDMs	histone demethylases
HP1	heterochromatin protein 1
LSD1	lysine-specific demethylase 1
PCNA	proliferating cell nuclear antigen
phH2AX Ser139	histone H2AX phosphorylated on serine 139
pTRIPZ shRNA	empty lentiviral vector for doxycycline-inducible expression of an short hairpin RNA together with TurboRFP
