# Supplementary material for: Silencing Lysine-Specific Histone Demethylase 1 (LSD1) Causes Increased HP1-Positive Chromatin, Stimulation of DNA Repair Processes, and Dysregulation of Proliferation by Chk1 Phosphorylation in Human Endothelial Cells
Source: Cells. 2019 Oct 7;8(10):1212. doi: 10.3390/cells8101212 (PMC6829479; doi:10.3390/cells8101212)
Supplement: Supplementary file 1 [file cells-08-01212-s001.pdf]

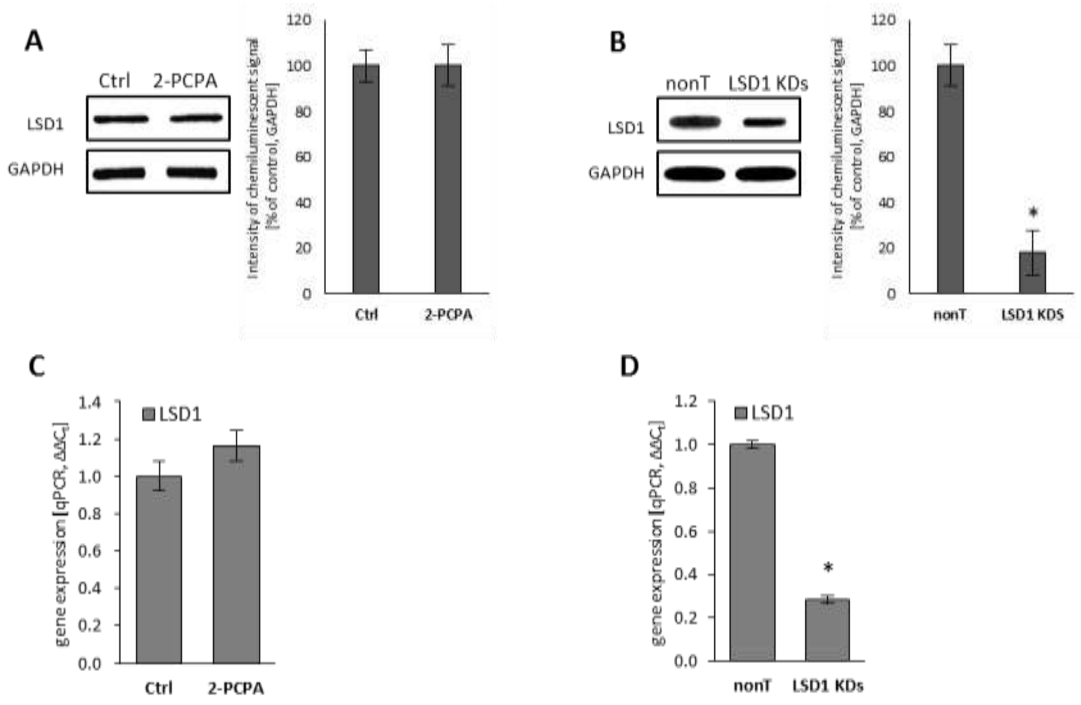

Figure_S.1. Expression level of LSD1 at the protein (upper images, charts; A, B) and transcript level (bottom graphs; C, D), modified by 2-PCPA treatment and due to shRNA silencing. Presented blots are representative of three independent experiments. Expression of LSD1 was estimated at least in three repeats and in case of nonT/Knockdown cells couple, every time when fresh set of knockdown cells was prepared. The data are presented as mean ± SD, *p<0.05, ANOVA and *post hoc* analysis by Tukey's test.

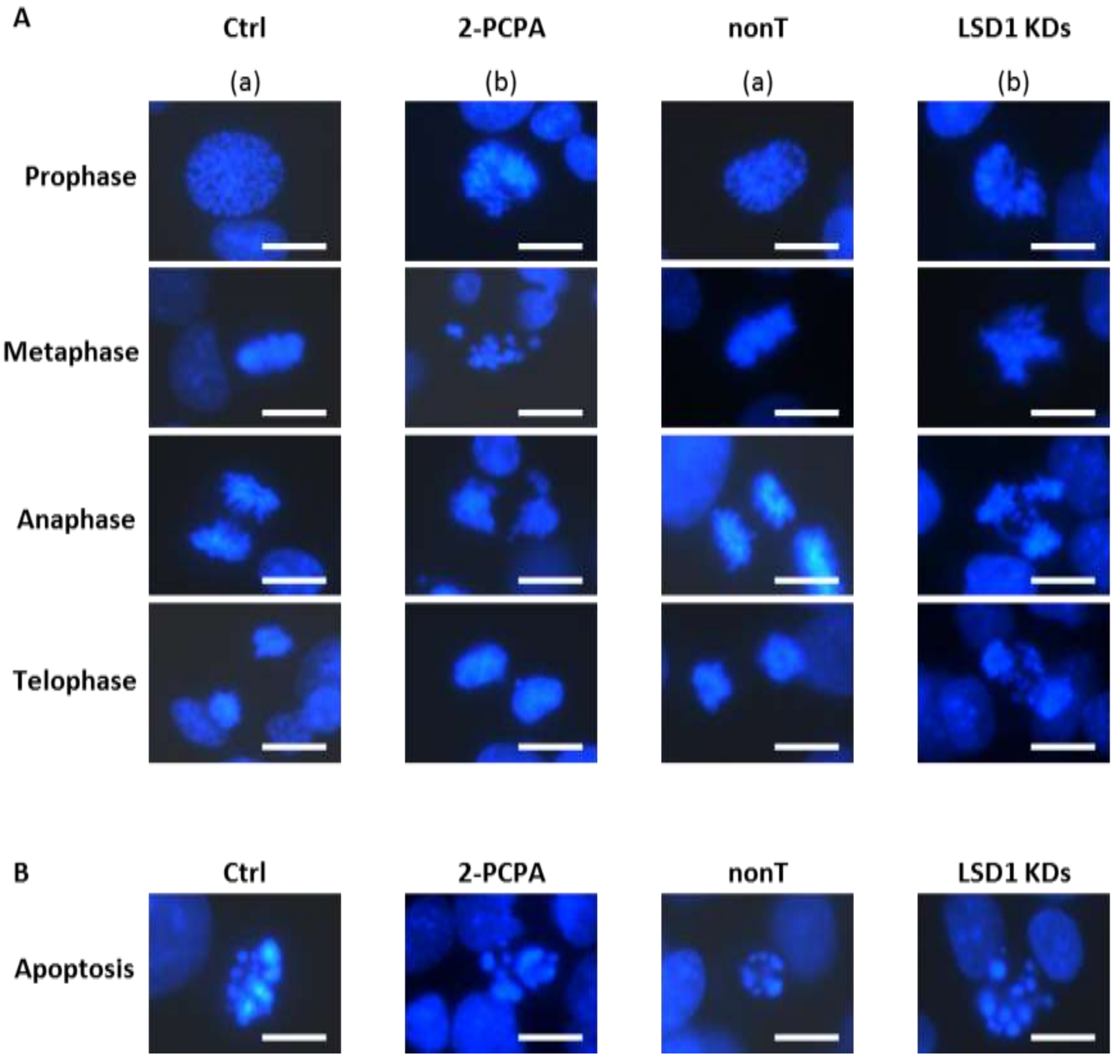

Figure_S.2. Visualization of cells in different phases of mitosis (A) and cells undergoing apoptosis (B) after LSD1 shutting down. Control cells for pharmacological (Ctrl) and transcriptional (nonT) of LSD1 silencing show normal mitosis (a), whereas cells treated with 2-PCPA/ shRNA present aberrant M phase. Scale bars = 10 µm.

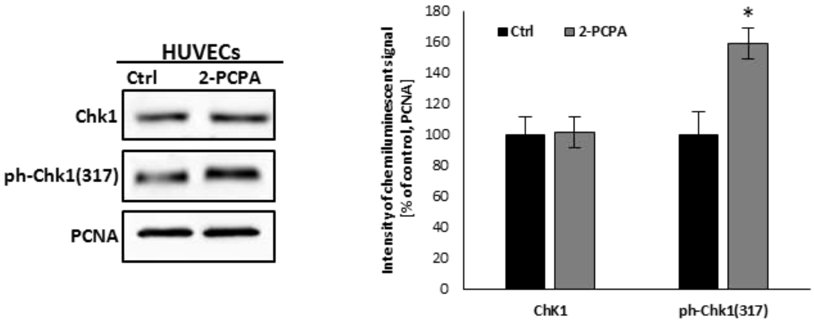

Figure_S.3. Effect of treatment of HUVECs with 2-PCPA on Chk1 activation. HUVECs at passage 3-5 were treated by 24 h with 100 µM 2-PCPA and whole cells extract was probed with specific antibodies to assess the level of expression of Chk1 and ph-Chk1(317). Presented blots are representative of three independent experiments. Chemiluminescence signal intensity assessment was performed by using ImageJ software. The data are presented as mean ± SD, *p<0.05, ANOVA and *post hoc* analysis by Tukey's test.

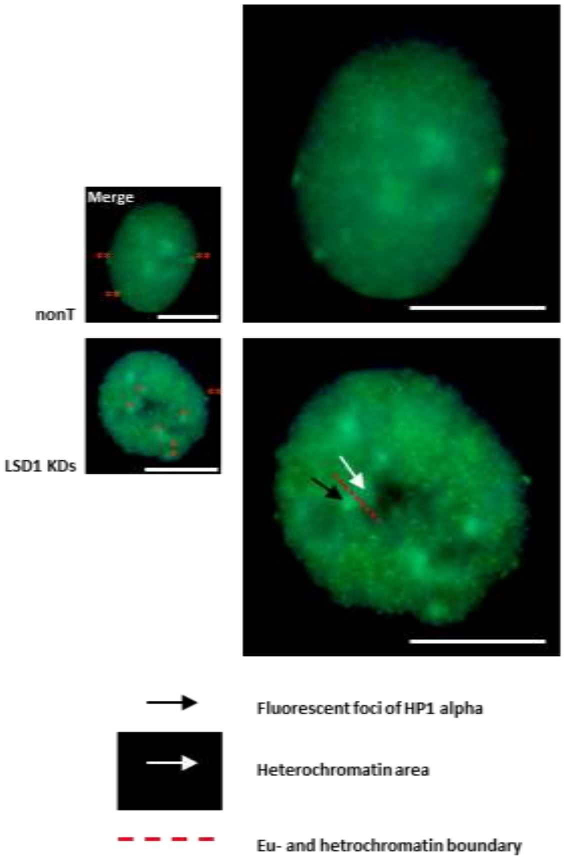

Figure_S.4. Visualisation of changes in the eu/heterochromatin fraction in HMEC-1 LSD1 KDs based on the HP1 alpha immunofluorescent staining. The presented images are magnified image of the Figure 3D. Scale bars are equal to 10 µm.

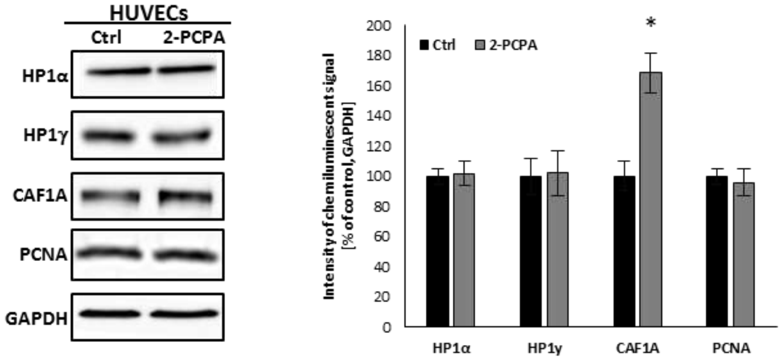

Figure_S.5. Effect of 2-PCPA on heterochromatin fraction remodeling in HUVECs. HUVECs at passage 3-5 were treated by 24 h with 100 µM 2-PCPA and whole cells extract was probed with specific antibodies to assess the level of expression of proteins characterizing heterochromatin 'topography': HP1 alpha, HP1 gamma, CAF-1A, PCNA. Presented blots are representative of three independent experiments. Chemiluminescence signal intensity assessment was performed by using ImageJ software. The data are presented as mean ± SD, *p<0.05, ANOVA and *post hoc* analysis by Tukey's test.

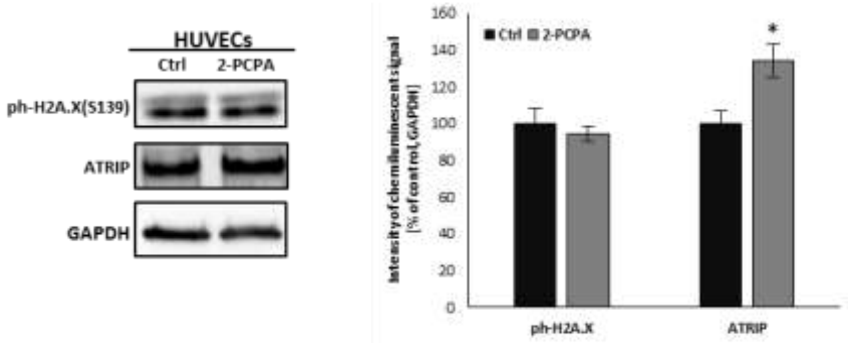

Figure_S.6. Effect of 2-PCPA on DNA damage indicators in HUVECs. HUVECs at passage 3-5 were treated by 24 h with 100 µM 2-PCPA and whole cells extract was probed with specific antibodies to assess the level of expression of proteins signalizing DNA damage. Presented blots are representative of three independent experiments. Chemiluminescence signal intensity assessment was performed by using ImageJ software. The data are presented as mean ± SD, *p<0.05, ANOVA and *post hoc* analysis by Tukey's test.
